# Operation for Ovarian Cyst in an Eleven Year Old Girl

**Published:** 1898-10

**Authors:** R. J. Alexander

**Affiliations:** Troy, Texas


// TEXAS MEDICAL JOURNAL.

ESTABLISHED JULY, 1885.

PUBLISHED MONTHLY.—SUBSCRIPTION $1.00 A YEAR.

VOL. XIV. AUSTIN, OCTOBER, 1898. No. 4.

### Original Contributions.

For the Texas Medical Journal.

### Operation for Ovarian Cyst in an Eleven Year Old Girl.

BY R. J. ALEXANDER, M. D., TROY, TEXAS.

Read before the Association of Physicians and Surgeons of Bell County, at Belton, Texas, August 16, 1898.

GENTLEMEN: In reporting this case I have nothing new to add to the technique of the operation, as most any man with surgical tact and training should safely perform the operation in one of our well regulated hospitals with the help of trained nurses and assistants; but we often have patients who are unable to go to an hospital to have an operation done.

This case demonstrates the fact that operation may with the proper care and precaution, be successfully performed outside of an hospital, as this one was done under the most unfavorable surroundings.

The operation should not be entered into without due consideration for and a thorough preparation of the patient. Ample provision for the management of complications should be made.

This patient is a mulatto girl 11 years of age. The history of this case dates back two months before operation. At this time the first notice was taken of an enlarged abdomen, and I was sent for and made diagnosis of ovarian cyst of right side The most remarkable features are: age of patient, size and rapidity in growth of tumor.

One week before operation this tumor had completely filled abdominal cavity, pressing against diaphragm, so that respiration was impaired, so much so, that to relieve the pressure I introduced a trocar into cyst and drew off about one gallon of fluid, which only relieved lower portion of enlargement.

I then decided I was dealing with a multilocular cyst and nothing would give relief but operative treatment. I at once begun preparation of patient for operation, which I did June 7, 1898, assisted by Drs. L. and L. F. Taylor, Claywell, Randolf and Nixon.

Every precaution possible was used in the preparation of patient, room, instruments, etc. Had patient to take a daily bath, scrubbing well with soap, for three days previous to operation. The evening before bichloride towels were applied to abdomen. Just before operation the abdomen and pubes were shaved and scrubbed with soap, warm water and a solution of permanganate of potash, lastly ending with a strong bichloride solution.

A free incision was made along median line from umbilicus to pubic bone. Finding my incision too small I enlarged it, cutting around navel, going two inches above. Then after breaking down what adhesions I could, I tried to bring tumor out through the opening, but found this impossible on account of its large size.

I found the cystic part of tumor occupying upper portion of abdominal cavity. I turned patient on her side, introduced trocar into one of the cysts, intending to empty them in this way, but patient could not breathe in this position, and I was compelled to put her on her back. Finding it would delay operation too long to empty the cysts with small trocar, and thinking the fluid non-septic, ruptured the cysts with my finger, emptying in this way four large cysts. I then succeeded in bringing tumor through the incision. The solid portion, weighing eight and one-half pounds, represented about one third of tumor. The cysts were of every variety of size, filled with what seemed to be a serous or sanguineous character of fluid, making it very difficult to classify. As the greater portion was cystic I classed it as a cystic tumor.

Immediately on getting the tumor through incision, assistants began pouring hot water into abdominal cavity, which controlled the oozing of blood.

I then ligated and divided the pedicle in the usual way.

The omentum, in this case, was adhered to both cyst and abdominal walls, and in the removal received considerable injury, and portions of it were considerably conjested, caused from pressure received in the situation.

Thinking there would be danger of unfortunate adhesions in this condition, I tied off and removed as much of it as I could safely, cutting away all ragged masses.

I examined pedicle and adhesions, and found no oozing of blood. The cavity was well sponged out, and wound closed by separate sutures; first, peritoneum and fibrous structure of the linea alba was closed with cat gut sutures; lastly, superficial fascia and skin closed with silk worm gut sutures. Dressing was then applied, and held in place by a many-tailed bandage.

Patient held up well under operation. Came from under influence of anæsthetic without any vomiting. very little shock; gave one-thirtieth grain strychnine hypodermically. One hour after operation temperature was 99½ degrees, elevation caused, I thought, from the rush of blood, pressure being removed.

Urine drawn off six hours after operation, and repeated every eight or ten hours, until third day voided urine without use of catheter.

In twenty-four hours, allowed one ounce and a half each of milk and water. Second and third days there was some pain in bowels, caused from peristaltic action, which was controlled by giving one-eighth grain morphia hypodermically. Temperature continued about normal, except evening of the second day it was 100½ degrees.

Allowed patient nothing but a fluid diet for eight days. On fifth day she complained of bowels wanting to move. I had an enema of warm soap suds given, which passed back with some gas.

Morning of the eighth day, bowels moved well. The afternoon of the same day, I re-dressed wound, found everything clean and nice, dressing dry, not a drop of pus.

On tenth day, took dressing off and removed half of stitches; union was complete.

Would have taken all the stitches out, but could not depend on my instructions being carried out in regard to movement of patient. I removed all the stitches on the twelfth day. Patient sat up a little on thirteenth day. On fifteenth day she walked around some in house.

It is now two months since operation, and she enjoys good health.

